# Reliability of Behavioral and fNIRS Neural Responses: Assessments During Posture–Inhibitory Control Dual Tasking

**DOI:** 10.3390/neurosci7030054

**Published:** 2026-05-02

**Authors:** Wan-Chun Su, Tony George, Marc H. Bornstein, Thien Nguyen, Amir Gandjbakhche

**Affiliations:** 1 Eunice Kennedy Shriver National Institute of Child Health and Human Development (NICHD), National Institutes of Health, Bethesda, MD 20892, USA; wanchunsu@lsu.edu (W.-C.S.); marc.h.bornstein@gmail.com (M.H.B.); thien.nguyen4@nih.gov (T.N.); 2 School of Kinesiology, Louisiana State University, Baton Rouge, LA 70802, USA

**Keywords:** dual task, posture, inhibitory control, functional near-infrared spectroscopy, reliability, capacity sharing theory

## Abstract

Background: Posture–inhibitory control dual-tasking is critical for safe daily functioning. Although functional near-infrared spectroscopy (fNIRS), a non-invasive neuroimaging tool, is increasingly used to examine neural mechanisms of dual-tasking, its reliability across posture transitions remains unclear. This study examined the reliability of behavioral and neural measures during sit-to-stand transitions and explored the neural mechanisms underlying posture–inhibitory control dual-tasking. Methods: Eighteen healthy adults (M age 42.8, SE = 3.3) completed tasks with varying posture challenges (sitting, standing, and tandem stance) and inhibitory demands (no task, congruent, and incongruent). Cortical activation was measured using fNIRS and reassessed after a 1 min sit-to-stand task. Results: Under lower task demands, activation in prefrontal and pre/postcentral regions increased with greater postural and inhibitory load, whereas this pattern reversed under higher demands. Behavioral performance demonstrated poor-to-excellent reliability (ICC range: 0.24 to 0.95; |r| = 0.33–0.97), whereas fNIRS measures showed poor-to-good reliability following sit-to-stand transitions (ICC range: <0 to 0.72; |r| = 0.02–0.79). Exploratory analyses suggested a shift from under- to over-additive cortical activation after posture transitions. Conclusions: Findings support the Capacity Sharing Theory, suggesting that postural and cognitive tasks compete for shared resources. Additionally, our findings reveal variable reliability of behavioral and neural measures across posture transitions. Future studies should account for postural changes when interpreting behavioral and neural findings.

## 1. Introduction

Dual tasks, or multitasking, refer to the capacity to engage in multiple activities concurrently and constitute a vital skill for navigating daily activities [1]. Among various types of dual tasks, posture–cognitive dual tasks hold particular importance on account of their frequent association with safety issues. For example, commuters conversing on phones while balancing in a moving metro and motorcyclists making prompt decisions based on road conditions require executive functioning and attention allocation to either of the tasks. When engaging in two tasks concurrently, performance of either or both tasks can be facilitated (i.e., dual-task facilitation) or undermined (i.e., dual-task interference or dual-task cost) [2]. Various cognitive theories propose potential mechanisms underlying attention/cognitive processing during dual tasks, yet offer inconsistent explanations. For example, Bottleneck Theory asserts that tasks requiring similar cognitive processes or involving similar neural networks are processed sequentially, leading to delayed responses to both tasks [3]. Conversely, Cross Talk Theory posits that tasks with overlapping neural processing do not mutually interfere and may even facilitate performance for each task [4]. However, these two theories may have limited applicability to tasks requiring different neural processing. By contrast, Capacity Sharing Theory postulates that capacity in neural processing is limited. When performing tasks simultaneously, competition and redistribution of resources are required [5]. Recent advances in neuroimaging tools have opened new avenues for examining cognitive models and investigating the mechanisms underlying dual-task performance.

To date, functional Magnetic Resonance Imaging (fMRI) studies have yielded inconsistent findings, including patterns of over-additive, additive, under-additive, and a combination of cortical activation patterns during cognitive–motor dual tasks [2]. Research supporting over-additive effects has identified greater cortical activation (mainly over the prefrontal, parietal, and cerebellum regions) during dual tasks compared to the sum of the component single tasks, implying additional neural processing required to integrate motor and cognitive networks [6,7]. Conversely, studies supporting additive activation have reported changes in neural activity during dual tasks similar to the sum of single tasks, suggesting no additional regions are required to process dual tasks [8,9]. Lastly, under-additive studies have noted that cortical activation during dual tasks is less than the sum of single tasks, pointing to limited resources and the need for resource competition when performing dual tasks [10,11]. However, due to constraints of MRI scanners, the motor tasks used in these studies have mostly been confined to finger and ankle movements and so have failed to investigate naturalistic motor–cognitive dual tasks.

Functional near-infrared spectroscopy (fNIRS) is a non-invasive neuroimaging tool that measures hemodynamic responses similar to fMRI [12]. But unlike fMRI, fNIRS allows participants to maintain upright positions, making it ideal for studying naturalistic posture–cognitive dual tasks [13]. Previous fNIRS studies have reported increased prefrontal cortex activation during posture–cognitive dual tasks compared to single tasks among younger adults and individuals with better dual-task performance [14,15,16,17]. However, other studies that involve older populations (with and without cognitive impairment) have resulted in mixed findings, with instances of increased, unchanged, and decreased prefrontal cortex activation during dual versus single tasks [15,18,19]. For example, Marusic et al. (2019) found greater cortical activation over the left dorsal lateral prefrontal cortex during a tandem stance–arithmetic dual task compared to single tasks in both younger and older adults [15], whereas St George et al. (2021) reported a continuous increase in prefrontal cortex activation as balance challenges intensified during a dual-task among younger adults, but decreased activation among older adults when balancing challenges became too demanding [17]. Fewer studies have examined the motor cortex in dual-task paradigms [14,18]. Specifically, Fujita et al. (2016) reported increased activation in the supplementary motor area during dual-tasks when compared with single-task conditions [14], whereas Rosso et al. (2017) did not find significant dual-task effects in the motor regions [18]. Moreover, the majority of fNIRS studies have utilized arithmetic as the cognitive component in dual-task paradigms [15,16,17,19], a design feature that has limited generalizability to real-life scenarios. In contrast, inhibitory control, a core executive function involving the suppression of automatic responses, underlies many everyday decisions and provides a more ecologically relevant cognitive challenge [20]. The current study builds on that design advantage to simulate real-world dual-task demands. Here, participants maintain postures of varying difficulty while engaging in tasks requiring suppression of responses to stimuli.

Behavioral findings indicate good reliability of inhibitory control tasks, with test–retest reliability of reaction time ranging from 0.65 to 0.71 [21]. On the other hand, fNIRS has demonstrated acceptable test–retest reliability during motor (e.g., finger tapping), postural (e.g., weight shifting), and visual tasks (ICCs > 0.78) [22,23]. fNIRS measures have also shown significant correlations with behavioral outcomes and have been used to track intervention-related changes, suggesting that fNIRS is a valid and reliable tool capable of detecting clinically meaningful differences [24,25]. However, most fNIRS studies have been conducted under relatively sedentary conditions, and the reliability of neural measures following postural transitions remains largely unknown. In everyday life, individuals frequently transit between postures (e.g., rising from a chair to stand) prior to engaging in cognitive–motor tasks. Such transitions can alter cerebral blood flow, cardiovascular dynamics, and neural recruitment, potentially compromising the reliability of fNIRS signals [26]. Indeed, Mol et al. (2019) reported that forehead fNIRS measures were sensitive to postural changes, showing different responses when transitioning from sitting or supine positions to standing [26]. Evaluating the reliability of both behavioral and neural measures following sit-to-stand transitions is therefore critical for distinguishing dual-task-related neural activation from variability attributable to postural changes [27]. Establishing test–retest reliability will improve the interpretability of dual-task fNIRS findings and inform the design of future studies investigating neural mechanisms underlying ecologically valid cognitive–motor behaviors. Accordingly, the current study is aimed at examining neural mechanisms associated with posture–inhibitory control dual-tasking and at assessing the test–retest reliability of behavioral and fNIRS measures following sit-to-stand transitions. We hypothesized that cortical activation would increase with greater inhibitory and postural demands and that behavioral performance would demonstrate good reliability following sit-to-stand transitions, whereas neural measures would exhibit reduced reliability [16,26,28].

## 2. Materials and Methods

### 2.1. Participants

Twenty healthy volunteers were recruited. A power analysis for intraclass correlation coefficients (ICC) was conducted in R (version 4.5.2). Assuming a null ICC of 0.40 and an expected ICC of 0.70 with two repeated measurements, α = 0.05, and 80% power, a minimum of four participants was required. To ensure robustness against potential data loss, a larger sample was included in the study. Before study enrollment, eligibility assessments were conducted by a nurse practitioner through physical examinations. Inclusion criteria included individuals aged 18 to 65 with the ability to provide consent; exclusion criteria encompassed those with skin conditions hindering the wearing of an fNIRS cap, vascular conditions impeding sit-to-stand transitions, or any medical condition potentially affecting task performance. Among the initial participants, two were disqualified during screening (one due to a congenital heart disease, and the other due to high blood pressure). The final cohort comprised 18 participants (11 females, 7 males; M age 42.8 years, SE = 3.3 years, and range = 23–63 years old). All experimental procedures adhered to the study protocol approved by the Institutional Review Board (IRB) at the National Institute of Child Health and Human Development (NICHD protocol # 10CH0198) and principles outlined in the Declaration of Helsinki. Prior to study participation, written informed consent was obtained from all participants.

### 2.2. fNIRS Measurements

**Figure 1:**
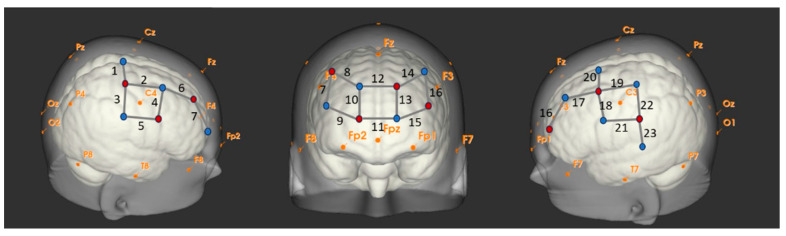
Placement of fNIRS probes (emitters marked in red; receivers in blue) and channel distribution (Channels #1 to #23) shown from left, frontal, and right perspectives. The orange font denotes landmarks based on the 10–20 system.

We used an NIRx fNIRS system (NIRScout system, NIRx Medical Technologies, LLC, New York, NY, USA), including 8 emitters and 11 receivers, to record hemodynamic changes across pre- and post-central regions (PCG) as well as the prefrontal (PFC) regions. The fNIRS probes were positioned bilaterally and anteriorly based on the International 10–20 system, with a spacing of 3 cm, resulting in 23 channels (Figure 1). The emitters emitted two wavelengths in the near-infrared spectrum (785 nm and 830 nm), penetrating the skull and creating a banana-shaped arc reaching the cortical area. The near-infrared light was scattered and absorbed by tissues before being detected by the receivers. Using the modified Beer–Lambert law, the attenuation of infrared light was used to calculate changes in concentrations of oxygenated (HbO_2_) and deoxygenated hemoglobin (HHb). When a cortical region is more active, an increase in HbO_2_ and a decrease in HHb are expected.

### 2.3. Experimental Procedures

**Figure 2:**
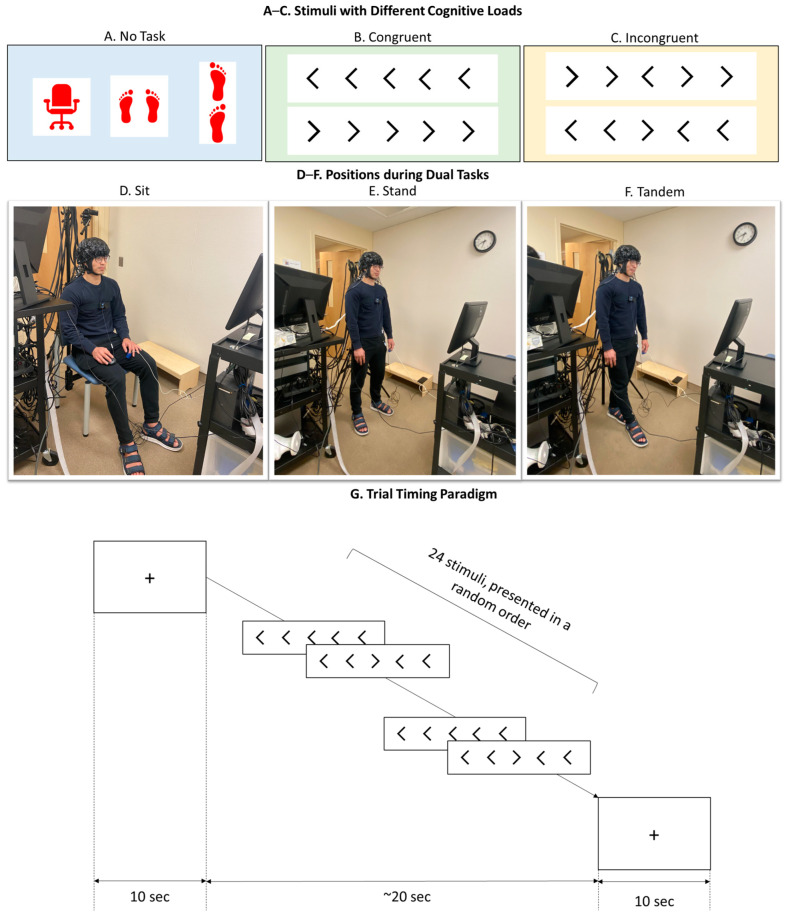
Picture examples for the posture–inhibitory control dual-task with different inhibitory demands (**A**–**C**), and balancing difficulties (**D**–**F**), as well as the trial-timing paradigm (**G**).

After being fitted with the fNIRS cap, participants were introduced to a dual-task paradigm combining cognitive and postural components. Inhibitory control was selected for the cognitive component, as it requires the suppression of automatic responses, promoting deliberate decision-making and enhancing safety. Participants’ inhibitory control performance was evaluated using the Flanker task [29], with stimulus presentation and trial marking managed through the E-prime system. The cognitive load, defined by varying levels of inhibitory demand, increased progressively across three conditions: (1) No Task: participants remained still and fixated on the stimuli (Figure 2A); (2) Congruent: five arrows pointing in the same direction were displayed, and participants responded based on the direction of the arrows (Figure 2B); and (3) Incongruent: five arrows were shown, with the central arrow pointing in a different direction than the flanking arrows. In this condition, participants were required to inhibit responses to the distracting flankers and respond only to the direction of the central arrow (Figure 2C). Participants were instructed to respond to the stimuli as rapidly as possible by clicking the left or right button of a wireless mouse, with reaction times recorded by the E-prime system.

For the postural component, participants performed the above-mentioned Flanker task in three positions that varied in balancing difficulties: Sitting (Figure 2D), Standing (Figure 2E), and Tandem positions (e.g., standing heel-to-toe in a straight line; Figure 2F). Tasks completed in a sitting position were considered single tasks due to their low demand on postural control. In contrast, tasks performed in standing and tandem positions were classified as dual tasks, with standing requiring lower postural demand and tandem stance imposing greater postural challenges. Balance difficulties increased sequentially from Sitting to Standing to Tandem positions. The order of task conditions was randomized using E-Prime.

Taken together, the current study comprised nine conditions varying in cognitive and postural demands: (1) sitting, no task; (2) sitting, congruent; (3) sitting, incongruent; (4) standing, no task; (5) standing, congruent; (6) standing, incongruent; (7) tandem stance, no task; (8) tandem stance, congruent; and (9) tandem stance, incongruent. Each condition included two trials, with each trial consisting of 24 stimuli and lasting approximately 20 s. Resting periods (10 s) were incorporated before and after the stimulus presentation, during which participants fixated on a central cross while remaining motionless. Please see the trial-timing paradigm in Figure 2G. Following the completion of the initial dual-task block, participants engaged in a 1 min sit-to-stand task, involving rapid alternation between sitting and standing within the 1 min time frame. This task is a responsive assessment that was used to quantify exercise capacity [30]. We chose this task because it frequently occurs in real-life situations, such as in office and school settings, and effectively mimics everyday postural transitions. The Borg Scale was employed to assess the participants’ perceived exertion during the sit-to-stand exercise [31]. On a scale of 10, the average score was 5.6 (standard deviation: 2.4), indicating that the sit-to-stand exercise is moderate intensity. After the sit-to-stand transitions, participants repeated the dual-task paradigm.

### 2.4. fNIRS Data Processing

We used Homer-3 (MGH—Martinos Center for Biomedical Imaging, Boston, MA, USA, version v1.87.0), an open-source MATLAB software (MathWorks, Inc., Natick, MA, USA, version R2017b), to estimate differences in the concentrations of HbO_2_ and HHb across conditions [32]. Our data processing pipeline was designed according to the “Best practices for fNIRS publications” [33]. First, we transformed attenuated light intensities into optical density. Subsequently, Principal Component Analysis (PCA) with a threshold set at 0.9 was employed to eliminate movement artifacts and reduce physiological noise [34]. Motion artifacts were identified using the hmrR_MotionArtifactByChannel function implemented in Homer3. This automated procedure detects artifacts at the channel level by applying a sliding window approach to the optical density time series and flagging segments that exceed predefined thresholds for signal amplitude and variability [32,35]. Specifically, we used the following parameters: motion window (tMotion) = 0.5 s, masking window (tMask) = 1.0 s, standard deviation threshold (STDEVthresh) = 20.0, and amplitude threshold (AMPthresh) = 0.20. Segments identified as motion artifacts were subsequently excluded or corrected using spline interpolation and Savitzky–Golay filtering methods [35]. We also performed low-pass filtering at 0.50 Hz to remove high-frequency noise, such as cardiac signals, and high-pass filtering at 0.01 Hz to remove low-frequency noise, such as data drift. Concentration changes in hemoglobin chromophores were calculated according to the modified Beer–Lambert Law [36], using individual differential pathlength factor (DPF) values determined via Equation (1) [37]. Hemodynamic response function (HRF) was achieved using a General Linear Model (GLM) of ordinary least squares [38], with the HRF shape modeled using a sequence of consecutive Gaussian functions [39]. The HRF time ranges were determined based on the experimental design, spanning from −10 s prior to, and 20 s after stimulation onset. In the current study, we focus on reporting HbO_2_ findings, as HbO_2_ is a measure more frequently documented in the fNIRS literature due to its superior signal-to-noise ratio [40]. Z-scores were calculated for HbO_2_ using Equation (2) [41,42] and averaged across the two trials. Lastly, outlier analyses were conducted to exclude trials with Z-scores greater or less than the averaged Z-score ± 2 standard deviations. A total of 9.8% of the fNIRS data were excluded:

(1) $$DPF\left(\lambda ,A\right)=\alpha +\beta A^{\gamma }+\delta \lambda^{3}+\varepsilon \lambda^{2}+\zeta \lambda$$

where *α* = 223.3, *β* = 0.5624, *A* = the age of the subject, *γ* = 0.8493, *δ* = −5.723 × 10^−7^, *λ* = Wavelengths (785 and 830), and *ε* = 0.001245; *ζ* = −0.9025:

(2) $$Zscore={(Mean}_{stim}-{Mean}_{baseline})/{SD}_{baseline}$$

### 2.5. Dual-Task Interference

In exploratory analyses, we calculated the differences in cortical activation between dual tasks and their component single tasks using Equation (3). A positive value indicates an over-additive, whereas a negative value indicates an under-additive cortical activation pattern:

(3) $$Dual\;Task\;Interference={Z\;score}_{Dual}-({Z\;score}_{Single1}+\;{Z\;score}_{single2})$$

### 2.6. Statistical Analyses

To analyze the accuracy and reaction times from the Flanker task, we conducted repeated-measures ANOVA with Posture (Sitting, Standing, and Tandem) and Condition (No task, Congruent, and Incongruent) as within-subject factors, and age and gender as covariates. For the assessment of fNIRS-related cortical activation, repeated-measures ANOVA was performed with Posture (Sitting, Standing, and Tandem), Condition (No-Task, Congruent, and Incongruent), and Regions (left PCG, left PFC, right PCG, and right PFC) as within-subject factors, and age and gender as covariates. Greenhouse–Geisser corrections were applied if the data violated the assumption of sphericity, as determined by Mauchly’s test. Post hoc analyses were conducted using paired *t*-tests with a significant *p*-value set at 0.05. To examine the reliability of the behavioral performance and cortical activation before and after the sit-to-stand transitions, we calculated the averaged-measure intraclass correlation coefficients (ICCs) using a two-way mixed-effects model with absolute agreement (ICC(A,1)) applied to trials averaged across the two pre- and post-sit-to-stand measurements. ICC values < 0.40 were interpreted as poor, values between 0.40 and 0.60 as fair, values between 0.60 and 0.75 as good, and values above 0.75 as excellent [43]. Supplementary Pearson correlation analyses were also performed to assess relative (rank-order) consistency across sessions. All statistical analyses were carried out using IBM SPSS software (SPSS, Inc., Chicago, IL, USA, version 31.0.2.0).

## 3. Results

### 3.1. Behavioral Performance During Posture–Inhibitory Dual Task (Accuracy and Reaction Time)

Response accuracy on the Flanker task was consistently high across postural conditions and levels of inhibitory demands, ranging from 95.44% to 98.83%. Accordingly, the two-way repeated-measures ANOVA examining the effects of Posture (Sitting, Standing, and Tandem) and Inhibitory Demand (Congruent, Incongruent) revealed no significant main effects or interactions for accuracy. In contrast, reaction time analyses showed a significant main effect of Posture (*F*(2, 26) = 4.60, *p* < 0.05). Post hoc pairwise comparisons indicated only a borderline difference in reaction times between Sitting and Standing postures (*t*(31) = 1.4, *p* = 0.177). Descriptive statistics, including means and standard errors (SE) for both accuracy and reaction time, are provided in Table 1.

### 3.2. Cortical Activation During Posture–Inhibitory Dual Task

**Figure 3:**
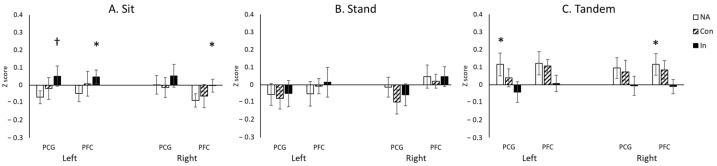
The effect of inhibitory demand on hemodynamic changes before sit-to-stand transitions. * indicates significant differences (*p* < 0.05); † indicates borderline significant differences (0.05 < *p* < 0.10).

Descriptive statistics for cortical activation, including the means and standard errors of the Z-scores, are provided in Table 2. The repeated-measures ANOVA revealed a significant main effect of Posture (*F*(2.0, 30.0) = 3.4, *p* < 0.05), a significant two-way interaction of Position × Condition (*F*(3.3, 48.7) = 5.7, *p* < 0.05), and a significant three-way interaction of Position × Condition × Region (*F*(12.0, 180.0) = 2.1, *p* < 0.05). Post hoc analyses showed significant effects of inhibitory demand on cortical activation. Specifically, the greatest cortical activations occurred in the Incongruent condition over bilateral PFC regions in the Sitting posture (*p*s < 0.05; Figure 3A) and in the Congruent condition over left PCG and right PFC in the Tandem posture (*p*s < 0.05; Figure 3C). No significant differences between conditions were observed in the Standing posture (*p*s > 0.05; Figure 3B).

**Figure 4:**
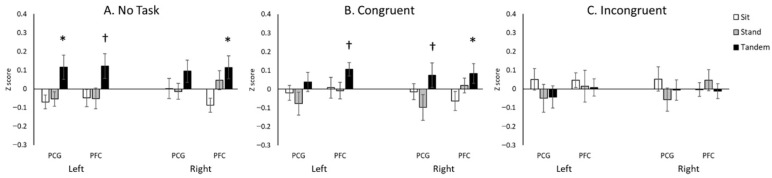
The effect of posture on hemodynamic changes before sit-to-stand transitions. * indicates significant differences (*p* < 0.05); † indicates borderline significant differences (0.05 < *p* < 0.10).

Regarding the effects of posture on cortical activation, greater activation was observed in the Tandem position compared to the other postures during the No-Task condition (over left PCG and right PFC) and the Congruent condition (over right PFC) (*p*s < 0.05; Figure 4A,B). No significant differences between postures were found in the Incongruent condition (*p* > 0.05; Figure 4C).

### 3.3. Reliability of Behavioral Performance and Cortical Activation After Sit-to-Stand Transitions

Overall, the behavioral and fNIRS measures demonstrated variable reliability following the 1 min sit-to-stand task. Based on ICC analyses, excellent reliability was observed for reaction time during the Tandem stance under the Incongruent condition (ICC = 0.95, 95% CI [0.86, 0.98]). Fair reliability was found during the Sitting Congruent (ICC = 0.47, 95% CI [−0.09, 0.80]), Standing Congruent (ICC = 0.54, 95% CI [0.08, 0.81]), and Standing Incongruent (ICC = 0.57, 95% CI [0.08, 0.83]) conditions, whereas poor reliability was observed during the Sitting Congruent (ICC = 0.24, 95% CI [−0.15, 0.62]) and Tandem Congruent conditions (ICC = 0.36, 95% CI [−0.19, 0.73]). Bland–Altman plots for all variables demonstrating fair-to-excellent ICCs are provided in Supplementary Figure S1. Pearson correlation analyses, reported as a supplementary index of relative (rank-order) reliability, yielded generally consistent results, with significant moderate-to-strong correlations observed during the Sitting Incongruent, Standing Congruent and Incongruent, and Tandem Congruent and Incongruent conditions (r = 0.66–0.97, *p*s < 0.05). In contrast, the reliability of cortical activation was more variable across conditions, ranging from poor to good. ICC analyses revealed poor reliability (ICCs < 0.4) across all ROIs during the Sitting Congruent, Standing Congruent and Incongruent, and Tandem Stance Congruent and Incongruent conditions. During the Sitting Incongruent condition, poor reliability was observed in the left PCG (ICC = 0.07, 95% CI [−0.28, 0.46]) and right PCG (ICC = 0.35, 95% CI [−0.08, 0.69]), fair reliability in the right PFC (ICC = 0.49, 95% CI [0.06, 0.77]), and good reliability in the left PFC (ICC = 0.72, 95% CI [0.40, 0.88]). Consistent with these findings, supplementary Pearson correlation analyses revealed a wide range of values (|r| = 0.02–0.79), with significant correlations observed only over bilateral PFC during the Sitting Incongruent condition (|r| = 0.59–0.75, *p*s < 0.05). Detailed ICC values, corresponding 95% confidence intervals, and Pearson correlation coefficients are provided in Table 3.

### 3.4. Exploratory Analysis of Cortical Activation Before and After Sit-to-Stand Transitions

**Figure 5:**
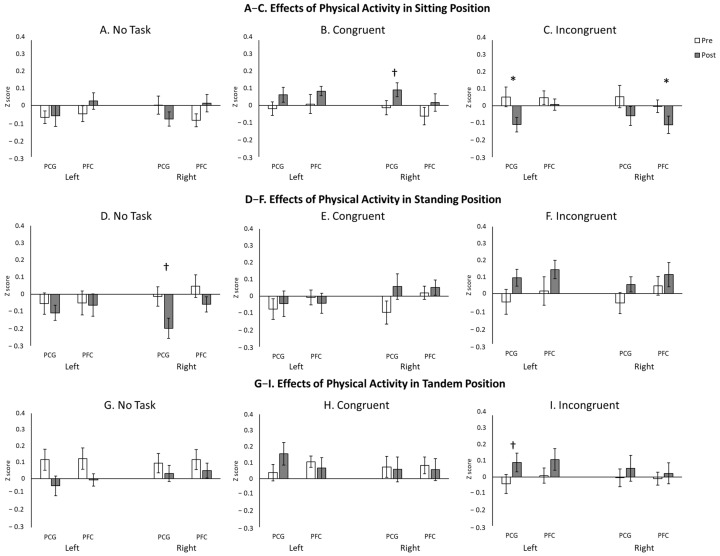
Exploratory analysis of hemodynamic changes before and after sit-to-stand transitions in sitting (**A**–**C**), standing (**D**–**F**), and tandem positions (**G**–**I**). * indicates significant differences (*p* < 0.05); † indicates borderline significant differences (0.05 < *p* < 0.10).

**Figure 6:**
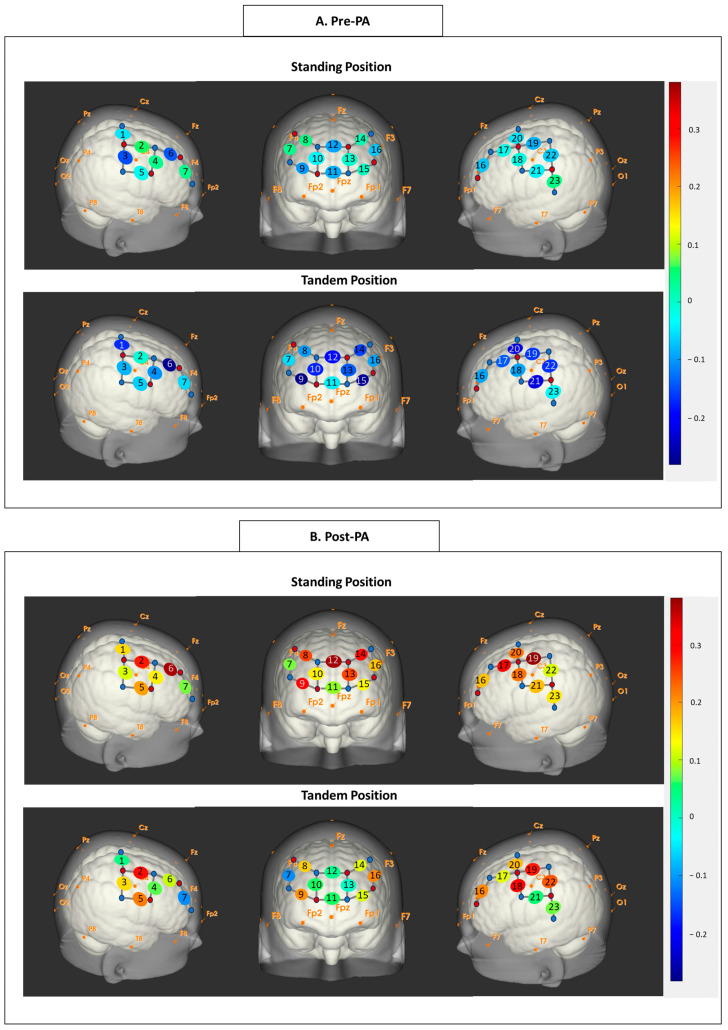
Dual-task interference before (**A**) and after the sit-to-stand transitions (**B**). The orange font denotes landmarks based on the 10–20 system.

Given the variable reliability of cortical activation measures, we conducted exploratory analyses to examine differences before and after the 1 min sit-to-stand task. In the Sitting position, a borderline increase in right PCG activation was observed during the Congruent condition (*p* < 0.10), whereas significantly reduced activation was found in the left PCG and right PFC during the Incongruent condition post-sit-to-stand compared to pre-sit-to-stand (Figure 5A–C). In the Standing position, a borderline decrease in right PCG activation was observed during the No-Task condition post-sit-to-stand compared to pre-sit-to-stand (Figure 5D–F). Lastly, in the Tandem position, a borderline increase in left PCG activation was found during the Incongruent condition post-sit-to-stand compared to pre-sit-to-stand (Figure 5G–I). Overall, the most pronounced changes in cortical activation following sit-to-stand transitions occurred in the Incongruent condition, which imposed the greatest cognitive workload. In addition, analysis of dual-task interference revealed a shift from an under-additive pattern (presented in blue) toward an over-additive pattern (depicted in red) from pre- to post-sit-to-stand (Figure 6). Paired *t*-tests confirmed significant increases in interference values over right PCG during the Standing, Incongruent condition and over the left PFC and PCG during the Tandem, Incongruent condition (*p*s < 0.05).

## 4. Discussion

Posture–inhibitory control dual-tasking is a critical daily function that is closely linked to safety. Despite its importance, cognitive models and neuroimaging findings regarding mechanisms underlying dual-task performance remain inconsistent, and no prior studies have examined the test–retest reliability of behavioral and neural measures across postural transitions. Accordingly, the current study investigated the effects of postural challenge and inhibitory control demand on dual-task performance as well as the test–retest reliability of neural measures following sit-to-stand transitions. Behaviorally, no significant differences were observed across levels of cognitive or postural demand, likely due to consistently high task performance. At the neural level, greater bilateral PFC activation was observed during higher inhibitory demands in seated conditions, whereas the opposite pattern emerged in the tandem stance, characterized by decreased left PCG and right PFC activation with increasing inhibitory demands. With respect to postural effects, greater left PCG and right PFC activation were evident in more challenging postures during the lower inhibitory demand condition, but not during the incongruent condition. Behavioral measures demonstrated test–retest reliability ranging from poor to excellent (ICCs = 0.24–0.95), whereas fNIRS measures showed variable test–retest reliability following sit-to-stand transitions. Exploratory analyses further suggested a shift from under-additive to over-additive cortical activation patterns after postural transitions. Collectively, these findings support the Capacity Sharing Theory and underscore the importance of accounting for postural transitions when interpreting neural responses during dual-task performance.

### 4.1. Effects of Inhibitory Demands and Postural Control on Behavioral Performance and Cortical Activation During Dual Tasks: An Under-Additive Pattern

Our behavioral findings showed no significant difference between tasks with varying cognitive and posture demands, probably due to the ceiling effect. In contrast, we observed differential cortical activation patterns corresponding to varying inhibitory demand and balance requirements; however, the patterns differed between simpler/single and more challenging/dual-task conditions. Specifically, in a less demanding posture (sitting), cortical activation increased with inhibitory demand, and in less cognitively demanding conditions (No task and Congruent conditions), cortical activation rose with balance requirements. These findings are consistent with previous fNIRS studies indicating greater PFC activation during posture–cognitive dual tasks compared to single posture or single cognitive tasks [14,16]. Together, these findings suggest that, when not reaching their limit, healthy adults were able to recruit greater cortical resources as their task complexity increased. However, during more complex tasks, we found no or even reversed cortical activation patterns during posture and inhibitory control dual tasks. In the Tandem position, activation in the left PCG and right PFC decreased as inhibitory demand increased. Moreover, during the Incongruent condition, no significant changes in cortical activation were observed with increasing postural challenges. Similar findings have been reported in previous fNIRS studies focusing on older adults, who normally find the dual tasks challenging [17,18]. For instance, in a posture–arithmetic dual-task, initial PFC activation increased among older adults with minor sensory posture modifications but decreased with greater sensory deprivation [17]. Our findings support the Capacity Sharing Theory, positing that under challenging dual-task conditions, posture and cognitive tasks need to compete for limited cognitive resources. In fact, our dual-task interference analyses evidenced an under-additive cortical activation pattern before sit-to-stand transitions, further supporting the Capacity Sharing Theory.

### 4.2. Acceptable Behavioral Test–Retest Reliability but Variable Neural Test–Retest Reliability Following Sit-to-Stand Transitions

Consistent with previous studies [28], we observed acceptable test–retest reliability across most behavioral measures (poor-to-excellent ICCs) following sit-to-stand transitions. For example, Szturm et al. (2017) reported moderate test–retest reliability (ICC = 0.60–0.65) for walking combined with a visuospatial executive functioning dual task [28]. In the present study, reaction time demonstrated fair-to-excellent reliability across most conditions, including sitting incongruent, standing congruent and incongruent, and tandem incongruent tasks (ICCs range = 0.47–0.95). In contrast, poor reliability was observed only for reaction time during the sitting congruent and tandem congruent conditions (ICCs = 0.24 and 0.36, respectively). Together, these findings suggest that behavioral measures may exhibit slightly better reliability under more cognitively demanding task conditions, possibly due to increased and more consistent engagement of executive control processes.

In contrast to the behavioral findings, fNIRS-related neural measures showed variable reliability following sit-to-stand transitions, highlighting a critical methodological challenge in interpreting cortical activation during posture–cognition dual tasks. Although acceptable test–retest reliability of fNIRS has been reported for well-controlled single-motor and postural tasks, such as finger tapping and weight shifting [22], the present results suggest that neural reliability is substantially reduced when assessments are separated by postural transitions. One contributor might be the subtle displacement of the fNIRS cap during movement. In fact, de Rond et al. (2023) demonstrated high test–retest reliability over the prefrontal cortex (ICC > 0.78) during finger tapping and postural tasks, which declined (to approximately 0.50) following cap removal and replacement [22]. It is therefore plausible that the repeated movement involved in the 1 min sit-to-stand task led to minor cap displacement, thereby reducing signal consistency across sessions. Reduced neural reliability may also be attributable to transient physiological changes associated with postural transitions, including alterations in cerebral blood flow and cardiovascular dynamics. Supporting this interpretation, Mol et al. (2019) showed that forehead fNIRS signals are highly sensitive to postural changes, with distinct hemodynamic responses observed when transitioning from sitting or supine positions to standing [26]. Together, these findings indicate that neural reliability may reflect a combination of measurement-related factors (e.g., optode displacement) and posture-induced physiological fluctuations. As such, careful consideration of postural transitions, additional physiological measures (e.g., heart rate, respiration), and quality assurance metrics (e.g., probe placement and contact quality) are essential when interpreting fNIRS data in ecologically valid dual-task paradigms.

### 4.3. Potential Influence of Short Bout of Physical Activities on Dual-Task Neural Activity (Methodological Consideration)

A methodological consideration of this study is that the 1 min sit-to-stand task may have functioned as a brief bout of physical activity, thereby influencing subsequent dual-task neural responses. Based on participant-reported Borg ratings, the sit-to-stand exercise in the current study was considered to be of moderate intensity. Acute physical activity is known to modulate executive functioning and dual-task performance and to alter associated cortical activation measured by fNIRS [44,45,46,47]. These effects may be mediated by increased cerebral blood flow and the release of neurotransmitters and neurotrophic factors, such as norepinephrine and dopamine [48,49,50]. Additionally, participants’ fitness levels may influence behavioral performance and neural activity during dual-task conditions [51]. In the current exploratory analyses, we observed changes in PFC and PCG activation following sit-to-stand transitions, with the most pronounced effects during the incongruent condition. Moreover, dual-task interference analyses revealed a shift from an under-additive activation pattern before sit-to-stand to an over-additive pattern after the transition. Together, the activity-induced modulations of neural responses may have contributed to variability in test–retest reliability and should be considered a methodological limitation when interpreting neural outcomes following postural transitions.

### 4.4. Limitations and Future Directions

The current study provides insight into the neural mechanisms underlying posture–inhibitory control dual-task performance while highlighting variable test–retest reliability of fNIRS measures following sit-to-stand transitions. However, it remains unclear whether this variability arises from optode displacement, posture-related physiological fluctuations, or the possibility that the sit-to-stand task itself functioned as a brief bout of physical activity that influenced subsequent dual-task performance. The absence of physiological monitoring (e.g., heart rate, respiration), information on participants’ fitness levels, and quality assurance metrics (e.g., probe contact quality) limited our ability to fully account for these effects. Future studies should incorporate additional control conditions or include physiological measures, physical activity questionnaires, and quality assurance metrics to disentangle these factors and better isolate sources of variability in neural measures. Since the differential pathlength factor (DPF) correction is commonly used in fNIRS data analysis, we did not assess its impact on our findings. Future studies should investigate how DPF affects the results. Additionally, this study did not incorporate structural MRI for precise spatial registration of the fNIRS probe layout, nor did it include quantitative assessments of postural control during the dual-task conditions. Future investigations should integrate spatial registration techniques and concurrent biomechanical measures of posture to improve anatomical specificity and to more directly link cortical activation patterns with behavioral and postural performance. Lastly, given the exploratory nature of the current study, we have reported borderline significant differences in cortical activations (*p*s < 0.10). However, readers should interpret these results with caution, and future studies with larger sample sizes are needed to verify these findings.

## 5. Conclusions

The current study provides support for the Capacity Sharing Theory, suggesting that tasks requiring distinct neural processing compete for limited resources when performed simultaneously. In addition, the findings revealed variable test–retest reliability of fNIRS neural measures following sit-to-stand transitions. fNIRS reliability may reflect a combination of optode displacement, posture-related physiological fluctuations, and the possibility that the sit-to-stand task itself functioned as a brief bout of physical activity that influenced subsequent dual-task performance. Notably, our exploratory analyses indicated a shift from under-additive to over-additive cortical activation patterns following postural transitions; however, these findings should be interpreted with caution given the study design. Future studies should incorporate additional control conditions, improved spatial registration methods, and concurrent assessments of postural control to better disentangle methodological, physiological, and task-related contributors to neural variability. Addressing these factors will be critical for improving the reliability and interpretability of fNIRS measures and for advancing our understanding of neural mechanisms underlying posture–cognitive dual-task performance in ecologically valid settings.

## Figures and Tables

**Table 1:** Means and SEs of the accuracy and reaction time during dual tasks before and after the sit-to-stand transitions.

	Accuracy		Reaction Time	
	*M*	*SE*	*M*	*SE*
Pre-Sit-to-Stand				
Sit, Congruent	98.70	0.50	427.26	31.35
Sit, Incongruent	95.44	3.19	453.47	19.38
Stand, Congruent	98.83	0.54	373.16	19.03
Stand, Incongruent	95.44	3.07	411.37	16.46
Tandem, Congruent	98.83	0.38	372.66	16.97
Tandem, Incongruent	96.09	3.10	411.61	11.91
Post-Sit-to-Stand				
Sit, Congruent	98.33	0.55	349.62	11.46
Sit, Incongruent	94.72	3.32	410.55	13.70
Stand, Congruent	99.17	0.45	352.41	13.38
Stand, Incongruent	94.44	3.32	391.27	20.49
Tandem, Congruent	98.89	0.49	353.09	18.35
Tandem, Incongruent	95.00	3.26	389.78	13.56

Note: Accuracy ranges from 0% to 100%; reaction time was greater than 250 ms.

**Table 2:** Means and SEs of Z scores for cortical activation before and after the sit-to-stand transitions.

	No-Task		Congruent		Incongruent	
	*M*	*SE*	*M*	*SE*	*M*	*SE*
Pre-Sit-to-Stand						
Sit, Left PCG	−0.07	0.04	−0.02	0.04	0.05	0.06
Sit, Left PFC	−0.05	0.05	0.01	0.06	0.05	0.04
Sit, Right PCG	0.00	0.05	−0.01	0.04	0.05	0.06
Sit, Right PFC	−0.09	0.04	−0.06	0.05	0.00	0.04
Stance, Left PCG	−0.05	0.06	−0.08	0.06	−0.05	0.07
Stance, Left PFC	−0.05	0.07	−0.01	0.04	0.01	0.09
Stance, Right PCG	−0.01	0.06	−0.10	0.07	−0.06	0.06
Stance, Right PFC	0.05	0.07	0.02	0.04	0.05	0.06
Tandem, Left PCG	0.12	0.06	0.04	0.05	−0.04	0.06
Tandem, Left PFC	0.12	0.07	0.11	0.04	0.01	0.05
Tandem, Right PCG	0.09	0.06	0.07	0.07	−0.01	0.05
Tandem, Right PFC	0.12	0.06	0.08	0.05	−0.01	0.04
Post-Sit-to-Stand						
Sit, Left PCG	−0.06	0.06	0.06	0.04	−0.11	0.04
Sit, Left PFC	0.03	0.05	0.08	0.03	0.01	0.03
Sit, Right PCG	−0.08	0.04	0.09	0.04	−0.06	0.06
Sit, Right PFC	0.01	0.05	0.02	0.05	−0.11	0.05
Stance, Left PCG	−0.11	0.04	−0.05	0.08	0.10	0.05
Stance, Left PFC	−0.06	0.07	−0.04	0.06	0.14	0.06
Stance, Right PCG	−0.20	0.06	0.06	0.08	0.05	0.05
Stance, Right PFC	−0.06	0.04	0.05	0.05	0.11	0.07
Tandem, Left PCG	−0.04	0.06	0.16	0.07	0.09	0.06
Tandem, Left PFC	−0.01	0.04	0.07	0.07	0.11	0.07
Tandem, Right PCG	0.03	0.05	0.06	0.08	0.05	0.08
Tandem, Right PFC	0.05	0.05	0.06	0.07	0.02	0.06

**Table 3:** Reliability of behavioral performance and cortical activation before and after the one-minute sit-to-stand task. ICCs, the 95% CIs, and Pearson correlations are reported.

Variables	ICC	95% CI Lower Bound	95% CI Upper Bound	Pearson Correlation
Behavioral Measures				
Reaction Time in Sitting, Congruent	0.24	−0.15	0.62	0.33
Reaction Time in Sitting, Incongruent	**0.47**	**−0.09**	**0.80**	0.72 **
Reaction Time in Standing, Congruent	**0.54**	**0.08**	**0.81**	0.83 **
Reaction Time in Standing, Incongruent	**0.57**	**0.08**	**0.83**	0.68 **
Reaction Time in Tandem, Congruent	0.36	−0.19	0.73	0.66 **
Reaction Time in Tandem, Incongruent	**0.95**	**0.86**	**0.98**	0.97 **
Cortical Activation				
Sitting, Congruent, Left PCG	−0.04	−0.45	0.42	−0.04
Sitting, Congruent, Left PFC	−0.49	−0.81	−0.02	−0.62 **
Sitting, Congruent, Right PCG	0.11	−0.29	0.51	0.12
Sitting, Congruent, Right PFC	−0.62	−0.89	−0.18	−0.61 **
Sitting, Incongruent, Left PCG	0.07	−0.28	0.46	0.09
Sitting, Incongruent, Left PFC	**0.72**	**0.40**	**0.88**	0.75 **
Sitting, Incongruent, Right PCG	0.35	−0.08	0.69	0.38
Sitting, Incongruent, Right PFC	**0.49**	**0.06**	**0.77**	0.59 **
Standing, Congruent, Left PCG	−0.06	−0.54	0.43	−0.05
Standing, Congruent, Left PFC	−0.17	−0.62	0.33	−0.17
Standing, Congruent, Right PCG	−0.24	−0.61	0.24	−0.25
Standing, Congruent, Right PFC	−0.06	−0.53	0.42	−0.06
Standing, Incongruent, Left PCG	−0.32	−0.67	0.15	−0.37
Standing, Incongruent, Left PFC	−0.38	−0.73	0.11	−0.417
Standing, Incongruent, Right PCG	−0.74	−0.95	−0.31	−0.79 **
Standing, Incongruent, Right PFC	−0.23	−0.65	0.27	−0.23
Tandem, Congruent, Left PCG	−0.02	−0.44	0.43	−0.02
Tandem, Congruent, Left PFC	0.02	−0.46	0.48	0.03
Tandem, Congruent, Right PCG	0.06	−0.44	0.52	0.06
Tandem, Congruent, Right PFC	0.12	0.38	0.56	0.12
Tandem, Incongruent, Left PCG	0.21	−0.21	0.59	0.23
Tandem, Incongruent, Left PFC	−0.04	−0.47	0.42	−0.04
Tandem, Incongruent, Right PCG	−0.18	−0.62	0.32	−0.18
Tandem, Incongruent, Right PFC	−0.41	−0.78	0.09	−0.43

Bold indicates fair-to-excellent reliability. ** indicates significant correlations (ps < 0.01).

## Data Availability

The data are not publicly available due to restrictions associated with participants’ privacy.

## Supplementary Materials

The following supporting information can be downloaded at: https://www.mdpi.com/article/10.3390/neurosci7030054/s1, Figure S1: Bland–Altman plots for all behavioral and neural variables demonstrating fair to excellent ICCs.

## References

[1] MacPherson S.E. (2018). Definition: Dual-tasking and multitasking. Cortex.

[2] Leone C., Feys P., Moumdjian L., D’Amico E., Zappia M., Patti F. (2017). Cognitive-motor dual-task interference: A systematic review of neural correlates. Neurosci. Biobehav. Rev..

[3] Pashler H. (1994). Dual-task interference in simple tasks: Data and theory. Psychol. Bull..

[4] Navon D., Miller J. (1987). Role of outcome conflict in dual-task interference. J. Exp. Psychol. Hum. Percept. Perform..

[5] Friedman A., Polson M.C., Dafoe C.G., Gaskill S.J. (1982). Dividing attention within and between hemispheres: Testing a multiple resources approach to limited-capacity information processing. J. Exp. Psychol. Hum. Percept. Perform..

[6] Van Impe A., Coxon J.P., Goble D.J., Wenderoth N., Swinnen S.P. (2011). Age-related changes in brain activation underlying single- and dual-task performance: Visuomanual drawing and mental arithmetic. Neuropsychologia.

[7] Wu T., Liu J., Hallett M., Zheng Z., Chan P. (2013). Cerebellum and integration of neural networks in dual-task processing. NeuroImage.

[8] Gruber O. (2001). Effects of domain-specific interference on brain activation associated with verbal working memory task performance. Cereb. Cortex.

[9] Johannsen L., Li K.Z., Chechlacz M., Bibi A., Kourtzi Z., Wing A.M. (2013). Functional neuroimaging of the interference between working memory and the control of periodic ankle movement timing. Neuropsychologia.

[10] Just M.A., Keller T.A., Cynkar J. (2008). A decrease in brain activation associated with driving when listening to someone speak. Brain Res..

[11] Rémy F., Wenderoth N., Lipkens K., Swinnen S.P. (2010). Dual-task interference during initial learning of a new motor task results from competition for the same brain areas. Neuropsychologia.

[12] Lloyd-Fox S., Blasi A., Elwell C.E. (2010). Illuminating the developing brain: The past, present and future of functional near infrared spectroscopy. Neurosci. Biobehav. Rev..

[13] McPartland J.C., Lerner M.D., Bhat A., Clarkson T., Jack A., Koohsari S., Matuskey D., McQuaid G.A., Su W.C., Trevisan D.A. (2021). Looking back at the next 40 years of ASD neuroscience research. J. Autism. Dev. Disord..

[14] Fujita H., Kasubuchi K., Wakata S., Hiyamizu M., Morioka S. (2016). Role of the frontal cortex in standing postural sway tasks while dual-tasking: A functional near-infrared spectroscopy study examining working memory capacity. BioMed Res. Int..

[15] Marusic U., Taube W., Morrison S.A., Biasutti L., Grassi B., De Pauw K., Meeusen R., Pisot R., Ruffieux J. (2019). Aging effects on prefrontal cortex oxygenation in a posture-cognition dual-task: An fNIRS pilot study. Eur. Rev. Aging. Phys. Act..

[16] Saraiva M., Fuentes-García J.P., Vilas-Boas J.P., Castro M.A. (2022). Relationship between physical activity level and sleep quality with postural control and hemodynamic response in the prefrontal cortex during dual-task performance. Physiol. Behav..

[17] St George R.J., Hinder M.R., Puri R., Walker E., Callisaya M.L. (2021). Functional near-infrared spectroscopy reveals the compensatory potential of pre-frontal cortical activity for standing balance in young and older adults. Neuroscience.

[18] Rosso A.L., Cenciarini M., Sparto P.J., Loughlin P.J., Furman J.M., Huppert T.J. (2017). Neuroimaging of an attention demanding dual-task during dynamic postural control. Gait Posture.

[19] Xu G., Zhou M., Chen Y., Song Q., Sun W., Wang J. (2024). Brain activation during standing balance control in dual-task paradigm and its correlation among older adults with mild cognitive impairment: A fNIRS study. BMC. Geriatr..

[20] Diamond A. (2013). Executive functions. Annu. Rev. Psychol..

[21] Lee S.H., Pitt M.A. (2024). Implementation of an online spacing flanker task and evaluation of its test-retest reliability using measures of inhibitory control and the distribution of spatial attention. Behav. Res. Methods.

[22] de Rond V., Gilat M., D’Cruz N., Hulzinga F., Orban de Xivry J.J., Nieuwboer A. (2023). Test-retest reliability of functional near-infrared spectroscopy during a finger-tapping and postural task in healthy older adults. Neurophotonics.

[23] Huang Y., Mao M., Zhang Z., Zhou H., Zhao Y., Duan L., Kreplin U., Xiao X., Zhu C. (2017). Test-retest reliability of the prefrontal response to affective pictures based on functional near-infrared spectroscopy. J. Biomed. Opt..

[24] Su W.C., Culotta M., Tsuzuki D., Bhat A. (2021). Movement kinematics and cortical activation in children with and without autism spectrum disorder during sway synchrony tasks: An fNIRS study. Sci. Rep..

[25] Su W.C., Tsuzuki D., Srinivasan S., Bhat A. (2024). Neural Effects of Creative Movement, General Movement, and Sedentary Play Interventions on Interpersonal Synchrony in Children with Autism Spectrum Disorder: A Preliminary fNIRS Study. Brain Sci..

[26] Mol A., Woltering J.H.H., Colier W.N.J.M., Maier A.B., Meskers C.G.M., van Wezel R.J.A. (2019). Sensitivity and reliability of cerebral oxygenation responses to postural changes measured with near-infrared spectroscopy. Eur. J. Appl. Physiol..

[27] Bornstein M.H., Putnick D.L., Esposito G. (2017). Continuity and stability in development. Child Dev. Perspect..

[28] Szturm T.J., Sakhalkar V.S., Kanitkar A., Nankar M. (2017). Computerized dual-task testing of gait and visuospatial cognitive functions; test-retest reliability and validity. Front. Hum. Neurosci..

[29] Eriksen C.W., Bundesen C., Shibuya H. (1995). The flankers task and response competition: A useful tool for investigating a variety of cognitive problems. Visual Selective Attention.

[30] Bohannon R.W., Crouch R. (2019). 1-minute sit-to-stand test: Systematic review of procedures, performance, and clinimetric properties. J. Cardiopulm. Rehabil. Prev..

[31] Borg G. (1998). Borg’s Perceived Exertion and Pain Scales.

[32] Huppert T.J., Diamond S.G., Franceschini M.A., Boas D.A. (2009). HomER: A review of time-series analysis methods for near-infrared spectroscopy of the brain. Appl. Opt..

[33] Yücel M.A., Lühmann A.V., Scholkmann F., Gervain J., Dan I., Ayaz H., Boas D., Cooper R.J., Culver J., Elwell C.E. (2021). Best practices for fNIRS publications. Neurophotonics.

[34] Cooper R.J., Selb J., Gagnon L., Phillip D., Schytz H.W., Iversen H.K., Ashina M., Boas D.A. (2012). A systematic comparison of motion artifact correction techniques for functional near-infrared spectroscopy. Front. Neurosci..

[35] Jahani S., Setarehdan S.K., Boas D.A., Yücel M.A. (2018). Motion artifact detection and correction in functional near-infrared spectroscopy: A new hybrid method based on spline interpolation method and Savitzky-Golay filtering. Neurophotonics.

[36] Kocsis L., Herman P., Eke A. (2006). The modified Beer-Lambert law revisited. Phys. Med. Biol..

[37] Nguyen T., Babawale O., Kim T., Jo H.J., Liu H., Kim J.G. (2018). Exploring brain functional connectivity in rest and sleep states: A fNIRS study. Sci. Rep..

[38] Ye J.C., Tak S., Jang K.E., Jung J., Jang J. (2009). NIRS-SPM: Statistical parametric mapping for near-infrared spectroscopy. Neuroimage.

[39] Diamond S.G., Huppert T.J., Kolehmainen V., Franceschini M.A., Kaipio J.P., Arridge S.R., Boas D.A. (2006). Dynamic physiological modeling for functional diffuse optical tomography. NeuroImage.

[40] Sato H., Fuchino Y., Kiguchi M., Katura T., Maki A., Yoro T., Koizumi H. (2005). Intersubject variability of near-infrared spectroscopy signals during sensorimotor cortex activation. J. Biomed. Opt..

[41] Ichikawa H., Nakato E., Igarashi Y., Okada M., Kanazawa S., Yamaguchi M.K., Kakigi R. (2019). A longitudinal study of infant view-invariant face processing during the first 3-8 months of life. NeuroImage.

[42] Su W.C., Dashtestani H., Miguel H.O., Condy E., Buckley A., Park S., Perreault J.B., Nguyen T., Zeytinoglu S., Millerhagen J. (2023). Simultaneous multimodal fNIRS-EEG recordings reveal new insights in neural activity during motor execution, observation, and imagery. Sci. Rep..

[43] Li L., Zeng L., Lin Z.J., Cazzell M., Liu H. (2015). Tutorial on use of intraclass correlation coefficients for assessing intertest reliability and its application in functional near-infrared spectroscopy-based brain imaging. J. Biomed. Opt..

[44] Moreau D., Chou E. (2019). The acute effect of high-intensity exercise on executive function: A meta-analysis. Perspect. Psychol. Sci..

[45] Park S.Y., Reinl M., Schott N. (2021). Effects of acute exercise at different intensities on fine motor-cognitive dual-task performance while walking: A functional near-infrared spectroscopy study. Eur. J. Neurosci..

[46] Talamonti D., Vincent T., Fraser S., Nigam A., Lesage F., Bherer L. (2021). The benefits of physical activity in individuals with cardiovascular risk factors: A longitudinal investigation using fNIRS and dual-task walking. J. Clin. Med..

[47] Vrinceanu T., Blanchette C.A., Intzandt B., Lussier M., Pothier K., Vu T.T.M., Nigam A., Bosquet L., Karelis A.D., Li K.Z.H. (2022). A comparison of the effect of physical activity and cognitive training on dual-task performance in older adults. J. Gerontol. B Psychol. Sci. Soc. Sci..

[48] Mehren A., Özyurt J., Lam A.P., Brandes M., Müller H.H.O., Thiel C.M., Philipsen A. (2019). Acute effects of aerobic exercise on executive function and attention in adult patients with ADHD. Front. Psychiatry.

[49] Mehren A., Özyurt J., Thiel C.M., Brandes M., Lam A.P., Philipsen A. (2019). Effects of acute aerobic exercise on response inhibition in adult patients with ADHD. Sci. Rep..

[50] Pontifex M.B., Saliba B.J., Raine L.B., Picchietti D.L., Hillman C.H. (2013). Exercise improves behavioral, neurocognitive, and scholastic performance in children with attention-deficit/hyperactivity disorder. J. Pediatr..

[51] Mekari S., Dupuy O., Martins R., Evans K., Kimmerly D.S., Fraser S., Neyedli H.F. (2019). The effects of cardiorespiratory fitness on executive function and prefrontal oxygenation in older adults. GeroScience.

