# Supplementary material for: Reliability of Behavioral and fNIRS Neural Responses: Assessments During Posture–Inhibitory Control Dual Tasking
Source: NeuroSci. 2026 May 2;7(3):54. doi: 10.3390/neurosci7030054 (PMC13214913; doi:10.3390/neurosci7030054)
Supplement: Supplementary file 1 [file neurosci-07-00054-s001.zip › neurosci-4165274-supplementary.pdf]

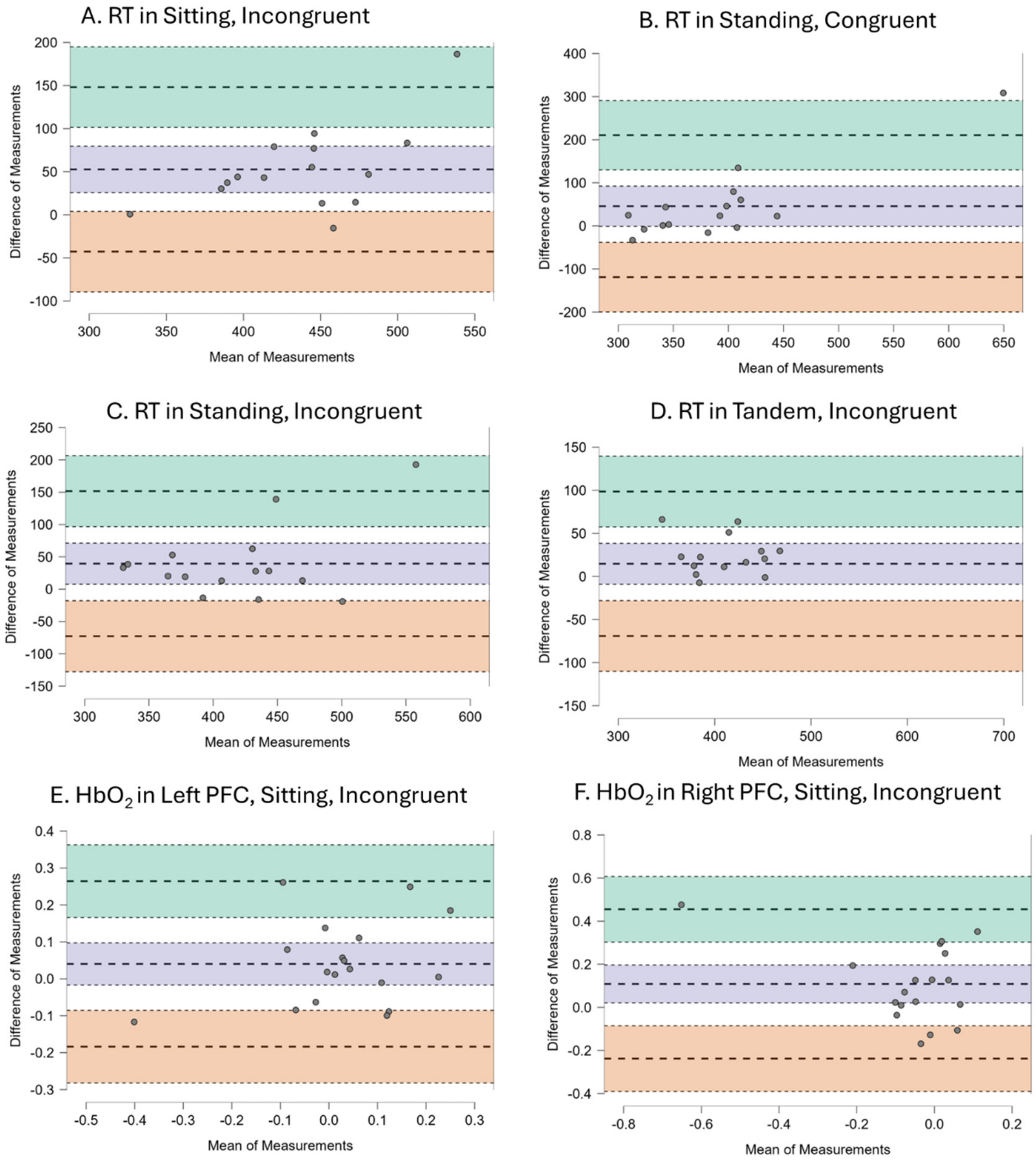

Figure S1. Bland–Altman plots for all behavioral and neural variables demonstrating fair to excellent ICCs.
